# Clinicopathological characteristics of mediastinal follicular dendritic cell sarcoma: report of three cases

**DOI:** 10.1186/s13019-016-0464-5

**Published:** 2016-04-11

**Authors:** Jibo Hu, Danjun Dong, Zhinong Jiang, Hongjie Hu

**Affiliations:** Department of Radiology, Sir Run Run Shaw Hospital, Zhejiang University School of Medicine, Hangzhou, Zhejiang 310016 China

**Keywords:** Follicular dendritic cell sarcoma, Clinicopathological characteristics, CT imagings, CD21, CD23

## Abstract

**Background:**

Follicular dendritic cell sarcoma (FDCS) is a rare malignant neoplasm of follicular dendritic cells that form a tight meshwork within lymphoid follicles. It occurs most commonly in the lymph nodes and occasionally at extranodal sites, but rarely in the mediastinum. It is an under-recognized clinical entity without clear diagnosis. Due to its rarity, FDCS is easily misdiagnosed by clinicians or pathologists.

**Case Presentation:**

Herein, we report three unusual cases of mediastinal FDCS, including one with hyaline vascular Castleman’s disease in woman. The clinicopathological characteristics and CT imagings were described. Their diagnosis was confirmed by immunohistochemical stainings of specific markers. Their therapeutic intervention, follow-up and outcomes were presented with brief literature review.

**Conclusions:**

A huge mediastinal shallowly-lobulated, demarcated soft tissue mass, with speckled, strip-like, coarse or arborizing calcification inside, and mild to moderate enhancement after contrast material administration on CT image, should consider FDCS as a possible diagnosis.

## Background

Follicular dendritic cell sarcoma (FDCS) is a rare malignancy arising from follicular dendritic cells which form an arborizing meshwork within lymphoid follicles [1]. Since Monda et al. first described the condition in 1986 [2], FDCS has become gradually known as the lymphatic and hematopoietic neoplasms based on the WHO classification [3]. Approximately 200 cases of FDCS have been reported in the English literatures so far. These lesions arise most frequently in lymph nodes and occasionally at extranodal sites, but rarely in the mediastinum [4]. Due to its uncommon systemic symptoms, FDCS is difficult to be diagnosed. Pathologically, FDCSs are well-circumscribed with spindle to ovoid cells arranged in a fascicular, whorled or storiform pattern. They typically present as a large mass with mean diameter ranging from 7 to 10 cm [5]. To the best of our knowledge, only three articles focused on the imaging features of FDCS [6–8]. Immunohistochemistry is required to confirm the diagnosis. FDCSs are usually positive for FDC markers CD21, CD23, and CD35 [9]. Tumor cells also typically express vimentin, a soft tissue sarcoma marker [10].

The current study presents three case of mediastinal FDCS and describes the pathological characteristics and chest CT imagings. The diagnosis of FDCS in these cases is confirmed by immunohistochemisty.

## Case Presentation

**Fig. 1:**
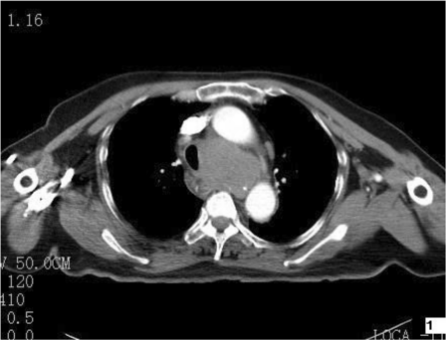
Axial contrast-enhanced CT image of the chest shows a well-defined lobulated homogeneous soft tissue mass in the superior middle mediastinum with peripheral speckle calcification and mild enhancement

Patient 1 was a 72-year-old woman. She complained about chest tightness, dyspnea and trachyphonia for 3 months. She had no fever, cough, expectoration or decompensation during the course of disease, except increased serum ferritin (663.6 μg/L). A contrast enhanced CT images of the chest revealed a well-defined mass of homogeneous attenuation in the superior middle mediastinum, and the mass measured 3.2 × 4.3 cm in diameter. An arborizing-pattern of coarse calcification was seen within the lesion (Fig. 1). The mass compressed adjacent trachea, esophagus and superior vena cava. It had similar homogeneous enhancement as the adjacent great vessels on 18-Fluoro-deoxyglucose positron emission tomography (FDG PET)-CT images. Thoracoscopic biopsy was then performed. The oval and fusiform tumor cells were slightly atypia with eosinophilic cytoplasm, and large nuclei with obvious nucleoli and mitoses. Mixture of lymphocytes and neutrophils patchily infiltrated among neoplastic cells. Immunohistochemical analysis showed that the tumor cells were positive with CD21, CD23, CD35 and HLA-DR, focal positive for CD20 and CD163. Patients received radiotherapy alone without surgery, and died of the disease due to metastasis one year after the diagnosis.

**Fig. 2:**
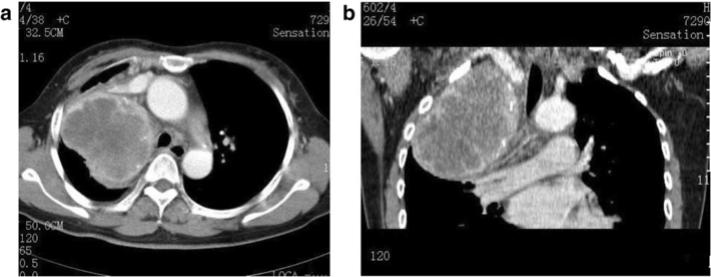
**a** Axial and (**b**) coronal contrast-enhanced CT images show a huge well-delineated shallowly-lobulated heterogeneously enhanced soft tissue mass in the right superior middle mediastinum with irregular hypodense areas of necrosis and multiple speckle-strip calcifications

Patient 2 was a 51-year-old woman presented with a history of chest tightness, chest pain, cough, expectoration and trachyphonia for 20 days. Serum ferritin was elevated to 234.4 μg/L. A contrast enhanced chest CT images revealed a well-defined 9.1 × 8.7 cm mass of homogeneous attenuation in the right superior middle mediastinum. A peripheral speckle-strip coarse calcification was seen within the lesion (Fig. 2a,b). The mass was excised and sectioned for histological examination. The plump spindle tumor cells were diffusely proliferated and densely arranged with significantly atypia, large nuclei and mitoses. Moreover, significant lymphocytic infiltration was noted. Immunohistochemical staining showed positive expression of CD 21 and CD35 in tumor cells. Patients underwent thoracic resection of the tumor and received radiotherapy after the surgery. Patient died of the disease due to metastasis 10 months after the diagnosis.

**Fig. 3:**
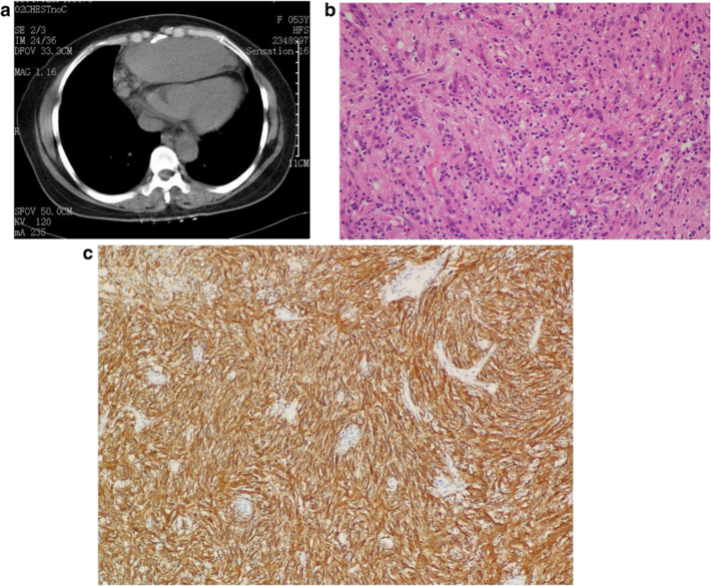
**a** Axial unenhanced CT image shows a well-demarcated shallowly-lobulated soft tissue mass in the anterior mediastinum, protruding to the left lung with several nodules around. **b** Photomicrograph (original magnification, ×200; hematoxylin-eosin stain) shows the oval and fusiform tumor cells, admixed with lymphocytes and neutrophils. **c** Photomicrograph shows CD-21 immunostaining positive tumor cells

Patient 3 was a 53-year-old woman who was admitted for a mediastinal mass found during annual physical examination. An unenhanced chest CT scan revealed a mass of 10.3 × 7.6 cm in the anterior mediastinum, with well-demarcated shallowly-lobulated soft tissue protruding to the left lung around several nodules (Fig. 3a). The entity was excised for histological examination. The tissue sections showed an intact capsule and fleshy pinkish white surface, bordering with the pericardium and bilateral mediastinal pleura, and several nodules around as well. Microscopically, the spindle tumor cells were in fascicular, swirling or diffusely irregular arrangement mixed with lymphocyte infiltration. Tumor cell nuclei were round, oval or fusiform, vacuolated with granular chromatin (Fig. 3b). Partial areas showed histological features characteristic of hyaline vascular Castleman’s disease. Moreover, the tumor cells were positive for CD21 (Fig. 3c) and CD35. The pathological diagnosis was mediastinal FDCS and hyaline vascular Castleman’s disease. Patient underwent surgical resection of the tumor without radiotherapy. Patient is still alive one and half years after surgery.

### CT imaging protocol

The Definition As CT scanner was utilized for Case 1, whereas the Siemens Sensation 16 CT scanner was applied for both Case 2 and 3. The single phase scanning method was used for the enhanced scan. Non-ionic contrast agent (iohexol or ioversol) of 90–100 mL was injected at a rate of 2.8–3.0 mL/s. Enhanced scan began 38 s after the injection of the contrast agent. The scan was performed with a thickness of 7 mm, a spacing of 7 mm, and a pitch of 1.0. Images were reconstructed with thickness of 2 mm.

## Discussion

According to the WHO classification of lymphatic and hematopoietic neoplasms, FDCS is grouped as the histiocytic and dendritic cell neoplasm [3]. FDCS equally affects males and females, with a median age of 47 years at diagnosis, widely ranged from 14 to 77 years [11]. The patients usually present with enlarged lymph nodes or a painless mass with a gradually increase in size. Systemic manifestations are rare. More than half of the lesions were located in lymph nodes, often involved in cervical and axillary nodes. Other cases were found in extranodal sites, including tonsils, spleen, oral cavity, parotid glands, gastrointestinal tract, liver, soft tissue, skin and even breast [12, 13].

The etiology and pathogenesis of FDCS have not been thoroughly clarified. Approximate 1/3 of cases in the literature were associated with Castleman’s disease, usually the hyaline vascular type, which may occur concomitantly or thereafter. Patients receiving long-term treatment are prone to develop this neoplasm [12].

Several pathologic features of FDCS have been described. The tumor cells usually are round, oval or spindle, with eosinophilic or amphophilic cytoplasm, small nucleoli, granular or vacuole chromatin, and mitosis. Focal storiform arrangement may be seen. The tumor cells are commonly interspersed with lymphocytes showing typically prominent perivascular cuffing. The tumor cells are typically positive for FDCS specific markers, such as CD21and CD35 [12,14]. Some also positively expressed Vimentin, S-100 and CD68 [15]. Similar to the previous reports, the tumor cells in this study are spindle, fascicular, swirl or diffusely arrangedwith lymphocyte infiltration. The tumor cells have fusiform and vacuolated nuclei with granular chromatin. The immunostaining results show positive expression of FDCS molecular markers, such as CD21 and CD35.

FDCS is extremely rare and scarcely reported in the radiological literature, which brought difficulty in making accurate diagnosis before operation or biopsy. According to our cases and those in the literature, mediastinal FDCSs locate in the anterior (see case 3), middle (see case 1 and 2) or posterior mediastinum [6,8], and are usually well-demarcated, slightly lobulated soft tissue masses sized from 3 cm to 10 cm (mean 6.7 cm). Necrosis and hemorrhage are uncommon, although areas of necrosis may be seen in huge tumors (see case 2). Peripheral calcifications are found in some cases, which are speckled, strip-like, coarse or arborizing. FDCS typically shows mild to moderate enhancement after contrast material administration. PET-CT and MRI may provide help to improve the diagnosis of the disease.

Mediastinal FDCS are different from Castleman’s disease, lymphoma and metastasis. Hyaline vascular Castleman disease classically shows much more intense contrast enhancement than FDCS. Calcification rarely occurs in untreated lymphoma or metastasis. A huge mediastinal mass with clear margin, internal coarse or arborizing calcifications, and relatively little necrosis or hemorrhage, favors imaging diagnosis of FDCS rather than other mediastinal tumors [6].

## Conclusions

A huge mediastinal shallowly-lobulated, demarcated soft tissue mass, with speckled, strip-like, coarse or arborizing calcification inside, and mild to moderate enhancement after contrast material administration on CT image, should consider FDCS as a possible diagnosis.

### Consent

Written informed consent was obtained from the patients for publication of this Case Report.

## Abbreviations

18-FDG-PET: 18-Fluoro-deoxyglucose positron emission tomography
CT: computerized tomography
FDCS: follicular dendritic cell sarcoma
HLA-DR: human leukocyte antigens-DR

## References

[1] Aguzzi A, Krautler NJ (2010). Characterizing follicular dendritic cells: A progress report. Eur J Immunol.

[2] Monda L, Warnke R, Rosai J (1986). A primary lymph node malignancy with features suggestive of dendritic reticulum cell differentiation. A report of 4 cases. Am J Pathol.

[3] Krokowski M, Merz H, Thorns C, Bernd HW, Schade U, Le Tourneau A (2008). Sarcoma of follicular dendritic cells with features of sinus lining cells--a new subtype of reticulum cell sarcoma?. Virchows Arch.

[4] Karligkiotis A, Contis D, Bella M, Machouchas N, Volpi L, Melis A (2013). Pediatric follicular dendritic cell sarcoma of the head and neck: a case report and review of the literature. Int J Pediatr Otorhinolaryngol.

[5] Krautler NJ, Kana V, Kranich J, Tian Y, Perera D, Lemm D (2012). Follicular dendritic cells emerge from ubiquitous perivascular precursors. Cell.

[6] Leipsic JA, McAdams HP, Sporn TA (2007). Follicular dendritic cell sarcoma of the mediastinum. AJR Am J Roentgenol.

[7] Kang TW, Lee SJ, Song HJ (2010). Follicular dendritic cell sarcoma of the abdomen: the imaging findings. Korean J Radiol.

[8] Long-Hua Q, Qin X, Ya-Jia G, Jian W, Xiao-Yuan F (2011). Imaging findings of follicular dendritic cell sarcoma: report of four cases. Korean J Radiol.

[9] Shinagare AB, Ramaiya NH, Jagannathan JP, Hornick JL, Swanson RS (2011). Primary follicular dendritic cell sarcoma of liver treated with cyclophosphamide, doxorubicin, vincristine, and prednisone regimen and surgery. J Clin Oncol.

[10] Aydin E, Ozluoglu LN, Demirhan B, Arikan U (2006). Follicular dendritic cell sarcoma of the tonsil: case report. Eur Arch Otorhinolaryngol.

[11] Hwang SO, Lee TH, Bae SH, Cho HD, Choi KH, Park SH (2013). Transformation of Castleman’s disease into follicular dendritic cell sarcoma, presenting as an asymptomatic intra-abdominal mass. Korean J Gastroenterol.

[12] Chan JK, Fletcher CD, Nayler SJ, Cooper K (1997). Follicular dendritic cell sarcoma. Clinicopathologic analysis of 17 cases suggesting a malignant potential higher than currently recognized. Cancer.

[13] Shia J, Chen W, Tang LH, Carlson DL, Qin J, Guillem JG (2006). Extranodal follicular dendritic cell sarcoma: clinical, pathologic, and histogenetic characteristics of an underrecognized disease entity. Virchows Arch.

[14] Biddle DA, Ro JY, Yoon GS, Yong YW, Ayala AG, Ordonez NG (2002). Extranodal follicular dendritic cell sarcoma of the head and neck region: three new cases, with a review of the literature. Mod Pathol.

[15] Clement P, Saint-Blancard P, Minvielle F, Le Page P, Kossowski M (2006). Follicular dendritic cell sarcoma of the tonsil: a case report. Am J Otolaryngol.

